# Hospital Acquired Infections in Surgical Patients: Impact of COVID-19-Related Infection Prevention Measures

**DOI:** 10.1007/s00268-022-06539-4

**Published:** 2022-04-06

**Authors:** Nicole Tham, Timothy Fazio, Douglas Johnson, Anita Skandarajah, Ian P. Hayes

**Affiliations:** 1 Colorectal Surgical Unit, The Royal Melbourne Hospital, Parkville, VIC Australia; 2 Department of General Surgical Specialties, The Royal Melbourne Hospital, Parkville, VIC Australia; 3 Department of Surgery, The Royal Melbourne Hospital, The University of Melbourne, Parkville, VIC Australia; 4 Department of General Medicine and Infectious Diseases, The Royal Melbourne Hospital, Melbourne, VIC Australia; 5 Department of Medicine, The Royal Melbourne Hospital, University of Melbourne, Parkville, VIC Australia; 6 Health Intelligence Unit, The Royal Melbourne Hospital, Parkville, VIC Australia

## Abstract

**Background:**

Hospital acquired infections are common, costly, and potentially preventable adverse events. This study aimed to determine the effect of the COVID-19 pandemic-related escalation in infection prevention and control measures on the incidence of hospital acquired infection in surgical patients in a low COVID-19 environment in Australia.

**Method:**

This was a retrospective cohort study in a tertiary institution. All patients undergoing a surgical procedure from 1 April 2020 to 30 June 2020 (COVID-19 pandemic period) were compared to patients pre-pandemic (1 April 2019–30 June 2019). The primary outcome investigated was odds of overall hospital acquired infection. The secondary outcome was patterns of involved microorganisms. Univariable and multivariable logistic regression analysis was performed to assess odds of hospital acquired infection.

**Results:**

There were 5945 admission episodes included in this study, 224 (6.6%) episodes had hospital acquired infections in 2019 and 179 (7.1%) in 2020. Univariable logistic regression analysis demonstrated no evidence of change in odds of having a hospital acquired infection between cohorts (OR 1.08, 95% CI 0.88–1.33, *P* = 0.434). The multivariable regression analysis adjusting for potentially confounding co-variables also demonstrated no evidence of change in odds of hospital acquired infection (OR 0.93, 95% CI 0.74–1.16, *P* = 0.530).

**Conclusion:**

Increased infection prevention and control measures did not affect the incidence of hospital acquired infection in surgical patients in our institution, suggesting that there may be a plateau effect with these measures in a system with a pre-existing high baseline of practice.

**Supplementary Information:**

The online version contains supplementary material available at 10.1007/s00268-022-06539-4.

## Introduction

### Background

Hospital acquired infections (HAI) are common adverse events in the developed world and remain challenging to address [1]. The reported incidence of HAI is 5–10%, increasing up to 30% for intensive care unit patients; the use of invasive devices (e.g. central lines) is associated with an increased risk [2, 3]. HAI prevention is a priority for reducing patient morbidity, mortality and costs.

In response to the COVID-19 pandemic, increased infection prevention and control (IPC) measures were implemented in hospitals to prevent transmission of COVID-19 to patients and staff. Among these measures was an emphasis on hand hygiene awareness. Hands are vectors for microorganism transmission; proper hand hygiene is the simplest intervention to reduce pathogen transmission and potential infection [4–6]. The effect of hand hygiene alone on HAI, however, is difficult to disentangle from the effect of concurrent IPC strategies [6–9]. In high-resourced healthcare settings, the impact of these IPC measures on HAI should be considered as a whole.

During the first half of 2020, Australia was in the unique position of being able to prepare for the COVID-19 pandemic without having suffered any significant COVID-19 impact on the hospital system, as there were small numbers of COVID-19 inpatient admissions and relatively low community transmission rates. This afforded us the opportunity to investigate the impact of the escalation in IPC behaviours on HAI in a low COVID-19 environment. We investigated outcomes in a single tertiary institution where changes in IPC strategies could be measured and substantiated.

### Objectives

The aim of this study was to determine the effect of COVID-19 pandemic-related escalation in IPC measures and hygiene behaviours on the incidence of HAI in surgical patients. We chose to study surgical patients surmising that they had a higher likelihood of requiring further minor invasive interventions where increased IPC measures might have effect. A secondary objective of this study was to investigate patterns of microorganisms involved in HAI pre- and during pandemic.

## Materials and methods

### Study design

This was a retrospective cohort study performed at The Royal Melbourne Hospital (RMH), Melbourne, Victoria, Australia. Ethics approval was granted by the institution (Study ID QA2021042). This study was reported in accordance with the STROBE statement [10].

### Participants

The exposure group was all admission episodes at RMH city campus with patients undergoing surgical interventions for the period 1 April 2020–30 June 2020. The comparison group was admission episodes with the same inclusion criteria pre-pandemic (1 April 2019–30 June 2019).

The time period for the exposure group corresponded with the first wave of COVID-19 in Victoria, Australia. There were 35 COVID-19 admission episodes relating to 32 patients within our institution in the study period. Daily case numbers within the state (population 6.6 million) were fewer than 100 per day [11].

Data were collected from several sources of administrative data, including patient administration system data, Australian Classification of Healthcare Interventions (ACHI) procedural codes and International Classification of Diseases, 10^th^ Revision, Australian modification (ICD-10-AM) codes.

### Exposure variables

The exposure of interest was the 2020 time period, during which there were hospital-wide changes in IPC measures and increased hygiene behaviours in response to the COVID-19 pandemic. These changes were substantiated from several sources. National Hand Hygiene Initiative (NHHI) audit data monitoring hand hygiene compliance were collected, audits were performed using intermittent sampling of ward staff by accredited nurses based on compliance with the WHO five moments of hand hygiene model [8]. The amount of alcohol-based hand sanitiser and universal disinfectant wipes procured for the hospital was measured and compared. Sales of personal scrubs from the hospital uniform shop were measured. Pre-pandemic, it was not routine for clinical staff to wear scrubs outside of the operating theatre. Hospital policies relevant to updates in hygiene behaviours such as social distancing and visitor restrictions were sourced from a hospital web-based platform.

### Measured co-variables

Details on patient demographics and comorbidities (using the Charlson Comorbidity Index (CCI) and the American Society of Anaesthesiologists (ASA) physical status classification system) were collected and categorised. Missing ASA score data were assigned a category of “Not recorded” to avoid data restriction in the final multivariable regression analysis. CCI was categorised into 0, 1 and ≥ 2, as any score ≥ 2 is associated with increased mortality. CCI has been validated for use in ICD-10-AM [12].

Admission data including elective or emergency admission, length of stay (LOS), and intensive care unit (ICU) or coronary care unit (CCU) admission were recorded. Procedural information of surgical unit subspecialty, anaesthetic type, duration of index operation, number of separate visits to the operating suite and ACHI codes for index procedure were recorded. Although LOS can be used as an outcome variable, in this study we have used it as an exposure variable. LOS was categorised as day case, overnight stay and > 2 days to discriminate between short and long stay patients and to act as an indirect measure of the extent of their procedure.

### Outcomes

The outcome variable “Hospital acquired infection” (HAI) was determined from a standardised list of hospital acquired complication (HAC) infection ICD-10-AM codes used by the Australian Commission on Safety and Quality in Health Care, with the indicator variable of not being present on admission [13]. Readmissions with HAI complications within 30 days of the index admission were also captured using administrative data.

HAI includes the diagnosis of urinary tract infections, surgical site infections (SSI), pneumonia, bloodstream infections, central line and peripheral line-associated bloodstream infection, multi-resistant organism, infection associated with prosthetic or implantable devices, gastrointestinal infection and other high impact infection (such as sepsis). Patients admitted with a pre-existing infection as their principal diagnosis on their index admission were excluded.

Clinical surveillance of HAI had been temporarily suspended during the pandemic study period. Clinical surveillance usually involved infection prevention and surveillance staff measuring and reporting on specific performance indicators such as central-line associated blood stream infections in the ICU, and SSI on specific patient cohorts.

The secondary outcome investigated was the pattern of microorganisms involved in HAI across both time periods. A file review was conducted of patients who had a HAI and microbiology results were correlated with their clinical notes and type of infection. The type and date of sample, organism cultured and antibiotic sensitivities were recorded.

### Statistical methods

Statistical analysis was performed using STATA version 16.0 (College Station, Texas, USA) statistical software. Baseline data were presented as counts, percentages, median values and inter-quartile range. Univariable logistic regression was used to analyse the odds of overall HAI for the principal exposure of interest (COVID-19 time period compared with pre-COVID-19) and for the other co-variables. The principal exposure of interest, and any co-variables with a greater than 10% effect on odds of HAI from the univariable regression were included in the multi-variable logistic regression analysis.

### Results

There was a total of 6208 admission episodes across the two time periods. After 263 admission episodes were excluded (listed index procedure unit was a medical unit), 5945 admission episodes were included in the study. There were 3415 admission episodes in the 2019 cohort and 2530 admission episodes in the 2020 cohort. Baseline patient demographics were similar across the two cohorts (Table 1), although there was a slightly higher proportion of patients in 2020 with a CCI of ≥ 2 (23.5% v. 20.7%). In terms of differences in operative admissions, there was a higher proportion of emergency admissions in 2020 (46.0% v. 40.3%) and fewer day stay cases (17.7% v. 20.7%). ICU or CCU admission involved 12.3% of patients in 2020 compared to 9.8% in 2019. Readmissions within 30 days for any HAI were similar across both cohorts (2.7% v. 2.8%). There were reductions in the number of breast, endocrine, head, neck and otolaryngology, and maxillofacial surgical procedures in 2020 (Online Resource 1). The number of procedures > 1 h and LOS > 2 days was recorded by surgical subspecialty and year of admission (Online Resource 2).

**Table 1:** Baseline demographics

	Pre-COVID Apr–Jun 2019	COVID Apr–Jun 2020	Total
Number of admissions	*n* = 3415 (57.4%), *n* (%)	*n* = 2530 (42.6%), *n* (%)	*n* = 5945 (100%), *n* (%)
Median Age (IQR)^1^ (years)	54 (36–68)	53.5 (36–68)	54 (36–68)
Age category (years)			
< 40	977 (28.6)	762 (30.1)	1739 (29.3)
40–59	1038 (30.4)	740 (29.2)	1778 (29.9)
≥ 60	1400 (41.0)	1028 (40.6)	2428 (40.8)
Sex Male: Female (Male%)	1981:1434 (58.0)	1525:1005 (60.3)	3506:2439 (59.0)
ASA score			
Low (ASA score 1–2)	1570 (46.0)	1100 (43.5)	2670 (44.9)
High (ASA score 3–5)	1193 (34.9)	941 (37.2)	2134 (35.9)
Not recorded	652 (19.1)	489 (19.3)	1141 (19.2)
Charlson Comorbidity Index			
0	2362 (69.2)	1668 (65.9)	4030 (67.8)
1	346 (10.1)	267 (10.6)	613 (10.3)
≥ 2	707 (20.7)	595 (23.5)	1302 (21.9)
Admission type			
Elective	2038 (59.7)	1365 (54.0)	3403 (57.2)
Emergency	1377 (40.3)	1165 (46.0)	2542 (42.8)
Anaesthetic type			
General Anaesthetic	2883 (84.4)	2067 (81.7)	4950 (83.3)
Regional/Sedation	224 (6.6)	180 (7.1)	404 (6.8)
Not recorded	308 (9.0)	283 (11.2)	591 (9.9)
Length of index operation (hours)			
≤1	1630 (47.7)	1064 (42.1)	2694 (45.3)
1–4	1362 (39.9)	1119 (44.2)	2481 (41.7)
> 4	265 (7.8)	224 (8.9)	489 (8.2)
Not recorded	158 (4.6)	123 (4.9)	281 (4.7)
ICU/CCU^2^ Admission Yes:No (Yes%)	336:3079 (9.8)	311:2219 (12.3)	647:5298 (10.9)
No. separate theatre visits			
1	3128 (91.6)	2264 (89.5)	5392 (90.7)
2	205 (6.0)	183 (7.2)	388 (6.5)
≥2	82 (2.4)	83 (3.3)	165 (2.8)
Length of stay			
Day case	707 (20.7)	448 (17.7)	1155 (19.4)
Overnight	808 (23.7)	592 (23.4)	1400 (23.5)
> 2 days	1900 (55.6)	1490 (58.9)	3390 (57.0)
Readmissions for hospital acquired infection	95 (2.8)	67 (2.7)	162 (2.7)
No. of episodes with repeat patient admission	394 (11.54)	218 (8.62)	612 (10.29)

*IQR*: interquartile range, *ICU*: intensive care unit, *CCU*: coronary care unit

### Infection prevention and control measures

During the 2020 study period, hand hygiene compliance increased from 82.8% (CI 81.8%–83.8%) in 2019 to 86.7% (CI 85.6%–87.7%) in 2020 (Table 2). Procurement of alcohol-based hand sanitiser doubled from 3178 to 6650L, and procurement of universal disinfectant wipes increased 1.8-fold. Sales of personal scrubs from the hospital uniform shop increased by 182%. Visitors were limited to 1 per patient per day for a period of 4 h. Additionally, social distancing was implemented (1.5 m between people). The use of face masks was not mandated in the treatment of non-COVID patients during this time period.

**Table 2:** Differences in infection prevention and control measures

	Pre-COVID Apr-Jun 2019	COVID Apr-Jun 2020
Hand hygiene compliance	82.8% (CI 81.8%—83.8%)	86.7% (CI 85.6%–87.7%)
Environmental cleaning measures		
Alcohol-based hand sanitiser procurement (litres)	3178	6650
Universal disinfectant wipe procurement (packets & tubs)	13,528	24,727
Other hygiene measures		
Visitor limitations	No limits	1 visitor per day for 4 h
Social distancing	None	1.5 m between people
Staff purchasing of surgical scrubs	–	Increased by 182%

N.B. The use of face masks was not mandated for treatment of non-COVID patients on the ward during the COVID time period studied

### Hospital acquired infections

There were 224 (6.6%) admission episodes associated with one or more HAI pre-pandemic and 179 (7.1%) during the 2020 pandemic period (Table 3). Univariable logistic regression analysis showed there was no evidence of change in the odds of having a HAI across the two time periods (Odds ratio (OR) 1.08, 95% confidence interval (CI) 0.88–1.33, *P* = 0.434) (Table 4). Multivariable logistic regression analysis showed no evidence of change in odds of acquiring a HAI comparing the two time periods (OR 0.93, 95% CI 0.74–1.16, *P* = 0.530), having adjusted for multiple potential confounding factors (Table 5).

**Table 3:** Number of hospital acquired infections by year of admission

	Pre-COVID Apr–Jun 2019	COVID Apr–Jun 2020	Total
	*N* = 3415 (57.4%), *n* (%)	*N* = 2530 (42.6%), *n* (%)	*N* = 5945 (100%), *n* (%)
No. of admission episodes with at least one hospital acquired infection	224 (6.6)	179 (7.1)	403 (6.8)
Urinary tract infection	44 (1.3)	41 (1.6)	85 (1.4)
Surgical site infection	52 (1.5)	43 (1.7)	95 (1.6)
Hospital acquired pneumonia	84 (2.5)	58 (2.3)	142 (2.4)
Bloodstream infection	15 (0.4)	11 (0.4)	26 (0.4)
Line-associated infection	4 (0.1)	0 (0.0)	4 (0.1)
Multi-resistant organism	37 (1.1)	35 (1.4)	72 (1.2)
Prosthetic-associated infection	28 (0.8)	25 (1.0)	53 (0.9)
Gastrointestinal infection	12 (0.4)	6 (0.2)	18 (0.3)
Other high impact infection	45 (1.3)	33 (1.3)	78 (1.3)

Percentages are out of total number of admission episodes per year

**Table 4:** Univariable logistic regression analysis: overall hospital acquired infection

		OR	95% CI	*P*-value
Admission year	2019	Ref		
	2020	1.08	0.88–1.33	0.434
Age category	40	Ref		
	40–59	1.51	1.10–2.08	0.011
	≥ 60	2.74	2.07–3.63	< 0.001
Sex	Male	Ref		
	Female	1.26	1.02–1.55	0.033
ASA score	Low (1–2)	Ref		
	High (3–5)	3.61	2.79–4.67	< 0.001
	Not recorded	2.80	2.07–3.78	< 0.001
CCI	0	Ref		
	1	2.17	1.60–2.96	< 0.001
	≥ 2	2.91	2.33–3.63	< 0.001
Admission type	Elective	Ref		
	Emergency	1.86	1.51–2.28	< 0.001
Anaesthetic type	Regional/sedation	Ref		
	General anaesthetic	2.37	1.32–4.26	0.004
	Not recorded	3.42	1.81–6.46	< 0.001
Length of index procedure (hours)	≤ 1	Ref		
	1–4	3.28	2.49–4.32	< 0.001
	> 4	7.12	5.10–9.96	< 0.001
	Not recorded	7.13	4.81–10.56	< 0.001
ICU/CCU Admission	No	Ref		
	Yes	7.28	5.84–9.06	< 0.001
No. of separate theatre visits	1	Ref		
	2	3.57	2.66–4.78	< 0.001
	> 2	9.27	6.58–13.08	< 0.001
Length of stay	Day case	Ref		
	Overnight	1.28	0.60–2.74	0.528
	> 2 days	12.94	7.07–23.65	< 0.001
Specialty unit	Plastic surgery	Ref		
	Breast & Endocrine	1.16	0.49–2.73	0.730
	Cardiothoracics	6.54	3.98–10.76	< 0.001
	Colorectal	2.98	1.60–5.54	0.001
	Head & Neck & Plastics	17.19	8.04–36.72	< 0.001
	Emergency General Surgery	1.93	1.12–3.31	0.017
	Head, Neck & Otolaryngology	1.03	0.44–2.43	0.941
	Hepatobiliary & Upper Gastrointestinal	2.68	1.31–5.47	0.007
	Nephrology Surgical	2.49	1.38–4.47	0.002
	Neurosurgery	2.45	1.48–4.05	0.001
	Oral & Maxillofacial	0.67	0.20–2.25	0.517
	Orthopaedic	3.44	2.18–5.41	< 0.001
	Thoracics	2.42	1.13–5.17	0.022
	Urology	1.49	0.82–2.70	0.193
	Vascular	3.85	2.18–6.80	< 0.001
No. of admission episodes per patient	1	Ref		
	> 1	1.98	1.51–2.59	< 0.001

*OR*: odds ratio, 95% *CI*: 95% Confidence interval, *ASA* score: American society of anesthesiology physical classification status score, *CCI* = Charlson comorbidity index, *ICU*: intensive care unit, *CCU*: coronary care unit, *Ref*: Referent

**Table 5:** Multivariable logistic regression analysis: overall hospital acquired infection

		OR	95% CI	*P*-value
Admission year	2019	Ref		
	2020	0.93	0.74–1.16	0.530
Age category (years)	40	Ref		
	40–59	1.22	0.85–1.74	0.275
	≥ 60	1.67	1.19–2.35	0.003
Sex	Male	Ref		
	Female	1.12	0.89–1.41	0.343
ASA score	Low (1–2)	Ref		
	High (3–5)	1.44	1.05–1.98	0.023
	Not recorded	1.38	0.93–2.05	0.106
CCI	0	Ref		
	1	1.37	0.97–1.95	0.074
	≥ 2	1.67	1.25–2.21	< 0.001
Admission type	Elective	Ref		
	Emergency	1.33	1.02–1.74	0.035
Anaesthetic type	Regional/sedation	Ref		
	General anaesthetic	1.13	0.60–2.16	0.701
	Not recorded	1.23	0.58–2.63	0.588
Length of index procedure (hours)	≤ 1	Ref		
	1–4	1.73	1.25–2.38	0.001
	> 4	2.24	1.45–3.46	< 0.001
	Not recorded	1.44	0.77–2.67	0.253
ICU/CCU Admission	No	Ref		
	Yes	3.71	2.76–4.99	< 0.001
No. of separate theatre visits	1	Ref		
	2	1.69	1.19–2.40	0.004
	> 2	4.16	2.65–6.53	< 0.001
Length of stay	Day case	Ref		
	Overnight	0.98	0.45–2.15	0.965
	> 2 days	3.62	1.85–7.08	< 0.001
Specialty unit	Plastic surgery	Ref		
	Breast & Endocrine	1.33	0.53–3.38	0.546
	Cardiothoracics	0.74	0.40–1.36	0.331
	Colorectal	2.63	1.31–5.30	0.007
	Head & Neck & Plastics	4.59	1.93–10.95	0.001
	Emergency General Surgery	1.19	0.65–2.17	0.574
	Head, Neck & Otolaryngology	1.03	0.41–2.61	0.938
	Hepatobiliary & Upper Gastrointestinal	1.67	0.74–3.77	0.218
	Nephrology Surgical	1.02	0.52–2.00	0.947
	Neurosurgery	1.00	0.57–1.76	0.993
	Oral & Maxillofacial	0.81	0.23–2.92	0.753
	Orthopaedic	2.03	1.22–3.36	0.006
	Thoracics	0.77	0.33–1.79	0.541
	Urology	1.31	0.68–2.53	0.424
	Vascular	0.97	0.51–1.83	0.925
No. of admission episodes per patient	1	Ref		
	> 1	2.35	1.72–3.21	< 0.001

*OR*: odds ratio, 95% *CI*: 95% Confidence interval, *ASA* score: American society of anesthesiology physical classification status score, *CCI*: Charlson comorbidity index, *ICU*: intensive care unit, *CCU*: coronary care unit. *Ref*: Referent

### Patterns of microorganisms

There were no major changes in the types of microorganisms involved in HAI across the two study periods. The percentages of the most common microorganisms involved in HAI across both time periods were similar (Table 6). Overall numbers were too small to conduct meaningful statistical analysis. Counts of multi-drug resistant organisms (MDRO) including Methicillin Resistant *Staphylococcus aureus* (MRSA) and Extended Spectrum Beta Lactamase (ESBL) *Escherichia coli* were similar across both time periods, and again too small for meaningful statistical analysis (Table 7).

**Table 6:** Most common microorganisms involved in hospital acquired infection

	Pre-COVID Apr–Jun 2019	COVID Apr–Jun 2020	Total
No. admission episodes with ≥ 1 HAI^1^	224	179	403
*S. aureus*	34 (15.2%)	26 (14.5%)	60 (14.9%)
MSSA^2^	25 (11.2%)	17 (9.5%)	42 (10.4%)
MRSA^3^	8 (3.6%)	8 (4.5%)	16 (4.0%)
*S. epidermidis*	12 (5.4%)	11 (6.1%)	23 (5.7%)
Enterococcus spp.	12 (5.4%)	15 (8.4%)	27 (6.7%)
*C. difficile*	10 (4.5%)	6 (3.4%)	16 (4.0%)
*E. coli*	33 (14.7%)	24 (13.4%)	57 (11.3%)
*P. aeruginosa*	17 (7.6%)	8 (4.5%)	25 (6.2%)
Klebsiella spp.	16 (7.1%)	15 (8.4%)	31 (7.7%)
No sample or no pathogen identified	89 (39.8%)	68 (38.0%)	157 (39.0%)

*HAI*: hospital acquired infection, *MSSA*: Methicillin sensitive *Staphylococcus aureus*, *MRSA*: Methicillin resistant *Staphylococcus aureus*

Percentages represent percentage out of total hospital acquired infection episodes (one episode may be associated with more than one type of infection)

**Table 7:** Multi-resistant organisms involved in hospital acquired infection

Multi-resistant organism	Pre-COVID Apr–Jun 2019				COVID Apr–Jun 2020				Total
Growth site	Wound or tissue	Urine	Other	Total	Wound or tissue	Urine	Other	Total	
*S. aureus* MRSA^1^	6	0	2	8	7	0	1	8	16
*S. epidermidis* Oxacillin resistant	5	0	1	6	6	0	0	6	12
*S. epidermidis* Multi-resistant	3	0	0	3	2	0	0	2	5
Enterococcus VRE^2^	1	1	0	2	1	0	2	3	5
*E. coli* ESBL	1	4	1	6	2	3	3	8	14
Klebsiella ESBL^3^	0	0	1	1	0	1	0	1	2

*MRSA*: Methicillin-resistant *Staphylococcus aureus*, *VRE*: Vancomycin resistant *enterococcus*, *ESBL*: Extended spectrum Beta-lactamase

### Discussion

This study found no evidence that the COVID-19 pandemic-related enhanced IPC measures and changes in hygiene-related behaviours were associated with change in the odds of HAI, despite adjusting for multiple potentially confounding variables including patient demographics, acuity of procedures and any changes in the proportion of surgical subspecialty workload across the two time periods (Table 5). There were no major changes in types of microorganisms involved, or in numbers of MDRO across the two time periods studied.

Few studies have investigated the impact of increased IPC measures during the COVID-19 pandemic on overall HAI specific to surgical patients. The studies that have shown decrease in infections related to COVID-19 pandemic response changes in IPC measures have been small and limited to SSI in specific cohorts of patients such as neurosurgery and cardiothoracic surgery [14, 15]. IPC measures were heterogenous and hand hygiene compliance rates were not consistently reported. An Italian study of general surgical patients found reduced rates of SSI, but was confounded by a very high proportion of breast and endocrine surgical cases in the COVID-19 era, which have low pre-existing risk of infection [16].

There are studies reporting the effect of enhanced IPC measures on HAI in hospital populations overall, rather than just surgical patients, with variable impact on HAI and MDRO. A Taiwanese study conducted in a low COVID-19 environment found no overall change in incidence density (incidence per 1000 patient days) of HAI. Although there was a reduction in incidence of Vancomycin-resistant enterococcus (VRE) and Carbapenem-resistant *Acinetobacter baumannii* (CRAB), no change in incidence was found for MRSA [17]. A study from a 1,785-bed hospital in Singapore found a decrease in hospital-acquired respiratory viral illnesses as well as decreased MRSA acquisition rates despite increased usage of broad-spectrum antibiotics. However, this study was performed in a moderately high COVID-19 environment without adjustments for patient demographics and other potentially confounding variables [18].

The impact of COVID-19 on HAI will vary based on the local burden of COVID-19 infections and the subsequent effect of that disease burden on the healthcare system. The ratio of COVID-19 to non-COVID-19 hospital admissions and other changes in the hospital system could potentially confound HAI outcomes. The suspension of mandatory reporting for HAI in many parts of the world during the height of the COVID-19 pandemic has made it difficult to accurately report on HAI outcomes during this period [19, 20]. The lack of change in HAI outcomes in this study cannot be attributed to major COVID-19 disease-related alterations in the hospital workflow and environment because the COVID-19 disease burden during the exposure time period was very low.

This was a rare opportunity to investigate the impact of increased IPC measures, including increased hand hygiene compliance, in a low COVID-19 environment. Hand hygiene compliance measurements based on the WHO 5 moments of hand hygiene are a standardised and observable process measure for quality improvement [6, 21]. However, hand hygiene auditing based on the WHO 5 moments is a relatively restricted metric and may not accurately reflect all changes in hand hygiene practices related to the COVID-19 pandemic. Although there was a modest increase in the sampled hand hygiene compliance in this study, the doubling of procurement of hand sanitiser suggests greater changes to overall hand hygiene practice. It should be noted that hand hygiene auditing is not equivalent to measuring alcohol-based hand rub usage and cannot assess changes or improvements in hand hygiene technique [9]. If the baseline rate of hand hygiene compliance is high and the overall rate of HAI is low, added improvements in hand hygiene compliance may not lead to any discernible effect on HAI. Additionally, the effect of hand hygiene alone is confounded by multiple concurrent infection prevention strategies. In the environment of this study, it was not possible to separate the effects of individual strategies; the changes in IPC measures were considered as a whole, with hand hygiene being one component. It is possible that incremental increases in IPC measures may not be able to modify the rate of HAI if a plateau effect is achieved.

### Limitations

This study is based on a retrospective review of administrative data to investigate HAI outcomes. The use of administrative data could under or over-ascertain HAI compared to traditional clinical surveillance methods, however, any inherent coding biases are likely to be non-differential over the two observed time periods. Coding practices for conversion of clinical data to administrative data in Australia are standardised and reliable [22].

The changes in IPC measures are described at a hospital-wide level and cannot be measured at an individual level. These results are from a single tertiary institution and may not be generalisable to other hospitals, however, this hospital is representative of Australian metropolitan tertiary hospitals.

### Conclusion

Australia was in a unique position amongst Western countries during the early phase of the COVID-19 pandemic to study the effects of increased IPC measures in a low COVID-19 environment. This study has found no evidence that increased hand hygiene compliance and other IPC measures altered the incidence of HAI in surgical patients despite adjusting for multiple confounding variables. Numbers of MDRO and overall types of microorganisms were similar throughout the two time periods. In a hospital environment with high levels of pre-existing infection prevention strategies including hand hygiene compliance, there may be a plateau in what further reduction in HAI can be achieved with the existing suite of IPC measures. Further reductions in HAI may require multiple sustained interventions. Given the morbidity and mortality associated with HAI, the need to find additional beneficial interventions is critical.

## Supplementary Information

Below is the link to the electronic supplementary material.

Supplementary file1 (PDF 53 KB)

Supplementary file2 (PDF 63 KB)
