# Supplementary material for: Hospital Acquired Infections in Surgical Patients: Impact of COVID-19-Related Infection Prevention Measures
Source: World J Surg. 2022 Apr 6;46(6):1249–58. doi: 10.1007/s00268-022-06539-4 (PMC8985564; doi:10.1007/s00268-022-06539-4)
Supplement: Supplementary file 1 — Supplementary file1 (PDF 53 KB) [file 268_2022_6539_MOESM1_ESM.pdf]

Online Resource 1. Procedural specialty by year of admission.

**Procedural specialty**	**Pre-COVID Apr – Jun 2019**	**COVID Apr – Jun 2020**	**Total**
	N = 3415 (57.4%), n (%)	N = 2530 (42.6%), n (%)	N = 5945 (100%), n (%)
**Breast and Endocrine Surgery**	140 (4.1)	74 (2.9)	214 (3.6)
**Cardiothoracic Surgery**	193 (5.7)	151 (6.0)	344 (5.8)
**Colorectal Surgery**	142 (4.2)	96 (3.8)	238 (4.0)
**Combined Head & Neck & Plastic Surgery**	25 (0.7)	17 (0.7)	42 (0.7)
**Emergency General Surgery**	319 (9.3)	283 (11.2)	602 (10.1)
**Head, Neck & Otolaryngology Surgery**	172 (5.0)	68 (2.7)	240 (4.0)
**Hepatobiliary & Upper Gastrointestinal Surgery**	112 (3.3)	54 (2.1)	166 (2.8)
**Nephrology Surgical**	209 (6.1)	132 (5.2)	341 (5.7)
**Neurosurgery**	370 (10.8)	322 (12.7)	692 (11.6)
**Oral & Maxillofacial Surgery**	113 (3.3)	44 (1.7)	157 (2.6)
**Orthopedic Surgery**	645 (18.9)	488 (19.3)	1133 (19.1)
**Plastic Surgery**	461 (13.5)	388 (15.3)	849 (14.3)
**Thoracic Surgery**	80 (2.3)	72 (2.9)	152 (2.6)
**Urology Surgery**	292 (8.6)	215 (8.5)	507 (8.5)
**Vascular Surgery**	142 (4.2)	126 (5.0)	268 (4.5)

Article title: Hospital acquired infections in surgical patients: impact of COVID-19-related infection prevention measures.

Journal name: World Journal of Surgery

Author names: Nicole Tham, Timothy Fazio, Douglas Johnson, Anita Skandarajah, Ian Hayes.

Corresponding author: Nicole Tham

Affiliations:

1. Colorectal Surgical Unit, The Royal Melbourne Hospital, Parkville, Victoria, Australia.
2. Department of General Surgical Specialties, The Royal Melbourne Hospital, Parkville, Victoria, Australia.
3. Department of Surgery, The Royal Melbourne Hospital, The University of Melbourne, Parkville, Victoria, Australia.

Corresponding author email address: nly.tham@gmail.com
