# Supplementary material for: Hospital Acquired Infections in Surgical Patients: Impact of COVID-19-Related Infection Prevention Measures
Source: World J Surg. 2022 Apr 6;46(6):1249–58. doi: 10.1007/s00268-022-06539-4 (PMC8985564; doi:10.1007/s00268-022-06539-4)
Supplement: Supplementary file 2 — Supplementary file2 (PDF 63 KB) [file 268_2022_6539_MOESM2_ESM.pdf]

Online Resource 2. Procedural specialty characteristics by year of admission.

	Pre-COVID Apr – Jun 2019			COVID Apr – Jun 2020		
**Specialty Unit**	**No. of patients**	**Case length >1hr, n (%)**	**LOS >2 days, n (%)**	**No. of patients**	**Case length >1hr, n (%)**	**LOS >2 days, n (%)**
**Breast and Endocrine Surgery**	140	48 (34.3)	30 (21.4)	74	37 (50.0)	24 (32.4)
**Cardiothoracic Surgery**	193	159 (82.4)	178 (92.2)	151	124 (82.1)	144 (95.4)
**Colorectal Surgery**	142	37 (26.1)	53 (37.3)	96	44 (45.8)	53 (55.2)
**Combined Head & Neck & Plastic Surgery**	25	24 (96.0)	25 (100.0)	17	14 (82.4)	16 (94.1)
**Emergency General Surgery**	319	142 (44.5)	200 (62.7)	283	125 (44.2)	152 (53.7)
**Head, Neck & Otolaryngology Surgery**	172	44 (25.6)	39 (22.7)	68	27 (39.7)	24 (35.3)
**Hepatobiliary & Upper Gastrointestinal Surgery**	112	77 (68.8)	46 (41.1)	54	39 (72.2)	28 (51.9)
**Nephrology Surgical**	209	71 (34.0)	103 (49.3)	132	57 (43.2)	71 (53.8)
**Neurosurgery**	370	283 (76.5)	308 (83.2)	322	273 (84.8)	268 (83.2)
**Oral & Maxillofacial Surgery**	113	48 (42.5)	39 (34.5)	44	20 (45.5)	17 (38.6)
**Orthopaedic Surgery**	645	363 (56.3)	430 (66.7)	488	320 (65.6)	332 (68.0)
**Plastic Surgery**	461	102 (22.1)	173 (37.5)	388	76 (19.6)	133 (34.3)
**Thoracic Surgery**	80	48 (60.0)	63 (78.8)	72	39 (54.16)	50 (69.4)
**Urology Surgery**	292	84 (28.9)	101 (34.6)	215	74 (34.4)	71 (33.0)
**Vascular Surgery**	142	97 (68.3)	112 (78.9)	126	74 (58.7)	107 (84.9)

Article title: Hospital acquired infections in surgical patients: impact of COVID-19-related infection prevention measures.

Journal name: World Journal of Surgery

Author names: Nicole Tham, Timothy Fazio, Douglas Johnson, Anita Skandarajah, Ian Hayes.

Corresponding author: Nicole Tham

Affiliations:

1. Colorectal Surgical Unit, The Royal Melbourne Hospital, Parkville, Victoria, Australia.

2. Department of General Surgical Specialties, The Royal Melbourne Hospital, Parkville, Victoria, Australia.
3. Department of Surgery, The Royal Melbourne Hospital, The University of Melbourne, Parkville, Victoria, Australia.

Corresponding author email address: nly.tham@gmail.com
